# Identification of diagnostic genes and drug prediction in metabolic syndrome-associated rheumatoid arthritis by integrated bioinformatics analysis, machine learning, and molecular docking

**DOI:** 10.3389/fimmu.2024.1431452

**Published:** 2024-07-29

**Authors:** Yifan Huang, Songkai Yue, Jinhan Qiao, Yonghui Dong, Yunke Liu, Meng Zhang, Cheng Zhang, Chuanliang Chen, Yuqin Tang, Jia Zheng

**Affiliations:** ^1^ Department of Orthopedics, People’s Hospital of Zhengzhou University, Henan Provincial People’s Hospital, Zhengzhou, Henan, China; ^2^ Department of Magnetic Resonance Imaging, The First Affiliated Hospital of Zhengzhou University, Zhengzhou, Henan, China; ^3^ Department of Immunology, College of Basic Medical Science, Dalian Medical University, Dalian, Liaoning, China; ^4^ Clinical Bioinformatics Experimental Center, People’s Hospital of Zhengzhou University, Henan Provincial People’s Hospital, Zhengzhou, Henan, China

**Keywords:** rheumatoid arthritis, metabolic syndrome, machine learning, molecular docking, immune infiltration

## Abstract

**Background:**

Interactions between the immune and metabolic systems may play a crucial role in the pathogenesis of metabolic syndrome-associated rheumatoid arthritis (MetS-RA). The purpose of this study was to discover candidate biomarkers for the diagnosis of RA patients who also had MetS.

**Methods:**

Three RA datasets and one MetS dataset were obtained from the Gene Expression Omnibus (GEO) database. Differential expression analysis, weighted gene co-expression network analysis (WGCNA), and machine learning algorithms including Least Absolute Shrinkage and Selection Operator (LASSO) regression and Random Forest (RF) were employed to identify hub genes in MetS-RA. Enrichment analysis was used to explore underlying common pathways between MetS and RA. Receiver operating characteristic curves were applied to assess the diagnostic performance of nomogram constructed based on hub genes. Protein−protein interaction, Connectivity Map (CMap) analyses, and molecular docking were utilized to predict the potential small molecule compounds for MetS-RA treatment. qRT-PCR was used to verify the expression of hub genes in fibroblast-like synoviocytes (FLS) of MetS-RA. The effects of small molecule compounds on the function of RA-FLS were evaluated by wound-healing assays and angiogenesis experiments. The CIBERSORT algorithm was used to explore immune cell infiltration in MetS and RA.

**Results:**

MetS-RA key genes were mainly enriched in immune cell-related signaling pathways and immune-related processes. Two hub genes (*TYK2* and *TRAF2*) were selected as candidate biomarkers for developing nomogram with ideal diagnostic performance through machine learning and proved to have a high diagnostic value (area under the curve, *TYK2*, 0.92; *TRAF2*, 0.90). qRT-PCR results showed that the expression of *TYK2* and *TRAF2* in MetS-RA-FLS was significantly higher than that in non-MetS-RA-FLS (nMetS-RA-FLS). The combination of CMap analysis and molecular docking predicted camptothecin (CPT) as a potential drug for MetS-RA treatment. *In vitro* validation, CPT was observed to suppress the cell migration capacity and angiogenesis capacity of MetS-RA-FLS. Immune cell infiltration results revealed immune dysregulation in MetS and RA.

**Conclusion:**

Two hub genes were identified in MetS-RA, a nomogram for the diagnosis of RA and MetS was established based on them, and a potential therapeutic small molecule compound for MetS-RA was predicted, which offered a novel research perspective for future serum−based diagnosis and therapeutic intervention of MetS-RA.

## Introduction

Rheumatoid arthritis (RA) is a chronic, systemic, autoimmune inflammatory disease that primarily affects the joints and soft tissue around the joints (1). RA patients have a high risk of developing cardiovascular diseases (CVD) and premature death due to systemic inflammation, which can shorten their expected lifespan by 5-10 years (2). Metabolic syndrome (MetS) is a common phenotype associated with increased CVD risk, including elevated fasting blood glucose, elevated triglycerides, low high-density lipoprotein, increased waist circumference, and hypertension (3). There are increasing evidences that RA is related to various components of MetS, such as weight changes, quantitative and qualitative dyslipidemia, characteristic adipokine profile, and insulin resistance, which increase CVD mortality (4). Previous research has established that MetS was prevalent in RA patients and that the risk of developing moderate to severe RA was higher in MetS patients than in those without MetS. Importantly, MetS-associated RA (MetS-RA) patients have higher disease activity than non-MetS-RA (nMetS-RA) patients (5), which was associated with the number of MetS components (6), suggesting that MetS may have an inflammatory environment that promotes the development of more severe RA.

Most recently, the interface between the metabolic system and the immune system has aroused great interest (7). Changes in immune-metabolic crosstalk contribute to the development of autoimmune diseases (8). Adipokines such as leptin, adiponectin, and lipoic acid-2 play multiple metabolic roles, which contributes to the onset of MetS and participates in the inflammatory process and immune regulation of RA (9). Consequently, exploring the molecular associations between RA and MetS is of great clinical value and research significance.

Currently, integrated bioinformatics analysis has been widely applied to identify new diagnostic genes and pathogenic genes related to various diseases (10, 11). Nevertheless, the genes for the common diagnosis of RA and MetS and the genes associated with each other are scarcely understood. Accordingly, the primary purpose of this study is to screen out the hub genes between RA and MetS, construct a diagnostic model, and provide new insights into the prevention and treatment of MetS-RA.

In this study, we used a variety of bioinformatics tools to search for MetS-RA hub genes by collecting RA datasets and MetS datasets from the Gene Expression Omnibus (GEO). Two MetS-RA hub genes (*TYK2* and *TRAF2*) were filtered by machine learning algorithms, and a diagnostic model for RA prediction was constructed. In addition, camptothecin (CPT), a potential small molecule compound for MetS-RA treatment, was discovered via connectivity map (CMap) analysis and molecular docking. We validated the expression of hub genes in fibroblast-like synoviocytes (FLS) from the synovium of MetS-RA patients and verified the effects of CPT on the cell migration and angiogenesis of MetS-RA-FLS *in vitro*. Finally, we explored the immune cell infiltration characteristics of RA and MetS.

## Materials and methods

### Microarray data collecting and processing

Three raw expression profile datasets of RA and healthy control groups, including GSE7307, GSE77298, and GSE206848, were obtained from the GEO database (https://www.ncbi.nlm.nih.gov/geo/) (12). The raw expression profile dataset GSE98895 of peripheral blood mononuclear cells (PBMCs) in MetS patients was obtained from the GEO database. Detailed description of the datasets is shown in **Table 1**. The combat function of “inSilicoMerging” package in R software (version 4.3.3) was used to merge three RA datasets and eliminate batch effect (13, 14).

**Table 1:** Basic information of GEO datasets used in the study.

GSE series	Tissue	Organism	Sample size		Platform
			Control	RA	
GSE206848	Synovium	Homo sapiens	7	2	GPL570
GSE77298	Synovium	Homo sapiens	7	16	GPL570
GSE7307	Synovium	Homo sapiens	6	5	GPL570
			Control	MetS	
GSE98895	PBMC	Homo sapiens	20	20	GPL649

### Differentially expressed genes analysis

Sangerbox Tools (http://www.sangerbox.com/tool) as a user-friendly interface supports differential analysis and provides interactive customizable analysis tools, including various kinds of correlation analyses, enrichment analyses, weighted correlation network analysis (WGCNA) as well as some other common tools and functions (15). Sangerbox Tools was utilized to missing values completion, data standardization, and gene symbol conversion of the integrated RA dataset and MetS dataset. The DEGs of RA and MetS datasets were obtained through the “Limma” package in R software (16), and the *P* value < 0.05 was statistically significant. The expression patterns of DEGs were then visualized in the form of volcanic plots and heatmaps.

### Functional enrichment analysis

RA and MetS gene expression profile data were imported into Gene Set Enrichment Analysis (GSEA) software (version 4.3.1), and selected the Kyoto Encyclopedia of Genes and Genomes (KEGG) subset to evaluate the relevant pathways and molecular mechanisms. The DEGs were imported into Sangerbox Tools, KEGG and Gene Ontology Biological Process (GO-BP) enrichment analysis was completed based on the latest subset gene annotation. Absolute value of Normalized Enrichment Score (NES) > 1.5, Nominal *P* value (NP) < 0.05 and false discovery rate (FDR) < 0.05 were considered statistically significant. The results of enrichment analysis were visualized by Sangerbox Tools.

### Weighted gene co-expression network analysis

The MetS gene expression profile data was imported into Sangerbox Tools, and the Median Absolute Deviation (MAD) of each gene was calculated, and the top 50% of the genes with the smallest MAD were excluded. The “goodSamplesGenes” function of the “WGCNA” package in R software was applied to remove outlier genes and samples (17). Further, a scale-free co-expression network was constructed based on the one-step network construction function of the “WGCNA” package. The appropriate “soft” threshold power (β = 3) was used for co-expression of similarity to calculate adjacency. Then, dynamic tree cuts and hierarchical clustering were performed, and a topological overlap matrix (TOM) was created to group genes into modules by random colors, and a gene dendrogram was constructed using a TOM-based measure of phase dissimilarity and a minimum gene cluster size (n = 30), the sensitivity was set to 3, and modules with distances less than 0.25 were merged. After obtaining the module, the different module eigengenes (ME) were obtained according to the first principal component of the module expression, and the modular-trait relationship was evaluated in line with the association between ME and MetS diagnosis. The module with the most significant positive correlation between the module-trait relationship was screened to obtain the genes contained in the module. And the correlation between module membership (MM) and Gene Significance (GS) scores in the module was evaluated to illustrate the module significance (MS). Sangerbox Tools was used to visualize the WGCNA analysis.

### Machine learning

Candidate genes of MetS-RA were obtained by overlapping RA DEGs, MetS DEGs, and the most significant module genes of MetS. The expression profile data of these candidate genes in RA dataset were screened for potential hub genes through the least absolute shrinkage and selection operator (LASSO) algorithm of Sangerbox Tools based on R software “glmnet” package. The R software “survival” package was used to integrate RA diagnosis and gene expression profile data, and the prognostic significance of each gene was further evaluated by COX method, and *P* value < 0.05 was statistically significant. The risk score was computed by the mRNA expression of diagnostic biomarkers weighted by their corresponding coefficients via Sangerbox Tools. RiskScore = -0.1374*CAPN3 + 0.0413*CKAP4 - 0.1862*CNBP + 0.0114*H2AFY2 + 0.0143*KCNK12 - 0.0441*KIR2DS1 + 0.0249*PIK3CD + 0.1887*TRAF2 + 0.6682*TYK2 - 0.0810*UQCRB. Candidate genes were further screened and the forest map was drawn. The R software “random forest” package was also used to screen out potential hub genes, with the Increase in Mean Squared Error (%IncMSE) *P* value < 0.01. The overlapping genes screened by Lasso-cox regression analysis and Random Forest (RF) algorithm were defined as hub genes.

### The construction of nomogram, the assessment of diagnostic marker prediction model, and the evaluation of diagnostic models in the external cohort

The nomogram of hub genes was constructed using the “rms” package of R software. The area under receiver operating characteristic (ROC) curve was plotted to evaluate the performance of hub genes and the nomogram in the diagnosis of RA. The calibration curves, decision curve analysis (DCA) and clinical impact curve (CIC) were used to evaluate the validity of nomogram in predicting MetS-RA. The validity of the above nomogram was verified by using external GEO datasets. Nomogram models based on hub genes from RA (GSE97779) and MetS (GSE142401) datasets were constructed respectively, meanwhile ROC, calibration curves, DCA, and CIC were used to evaluate the validity of the nomogram.

### Protein-protein interaction network analysis and cluster analysis

The overlapping genes of RA DEGs and MetS DEGs were analyzed by the STRING database (https://www.stringdb.org) (18), with a medium confidence score of > 0.4. The PPI network was imported into the Cytoscape software (version 3.10.2) for visualization. Further, the Cytoscape molecular complex detection (MCODE) function was used to screen the gene cluster with the highest score for the next small molecule compound prediction.

### Connectivity map analysis

The up-regulated genes in the highest scoring gene clusters from the MetS-RA PPI network were analyzed by the CMap database (https://clue.io) to search for potential small molecule compounds. The top 10 small molecule compounds with the highest negative enrichment scores were obtained.

### Molecular docking

The protein crystal structure (pdb format) of hub genes was obtained from the RCSB Protein Data Bank (http://www.pdb.org/). The structure of the small molecule compounds (sdf format) was obtained from the Pubchem (https://pubchem.ncbi.nlm.nih.gov/), and was converted to a pdb format through OpenBabel software (version 2.4.1). The proteins and compounds in pdb format were imported into Autodock tools software (version 1.5.7) for deleting water and adding hydrogenation. All flexible keys of compounds were rotatable by default and saved in pdbqt format as docking ligands. Then the molecular docking process was completed (19). According to the binding energy of proteins and compounds (< -6 kcal/mol), the tightly bound small molecular compounds were screened out. The molecular docking results were visualized using PyMOL (version 3.0).

### Immune cell infiltration analysis

RA and MetS gene expression profile data were analyzed by Sangerbox Tools immunoinfiltration analysis function. The R software “CIBERSORT” package was used to calculate the proportion of 22 types of immune cells in each sample by Wilcoxon test (20), *P* value < 0.05 was considered statistically significant, and stacked histogram visualization was performed. Spearman’s correlation coefficient was employed to analyze the proportion of infiltrating immune cells, *P* < 0.05 was considered statistically significant, and heatmap was used for visualization.

### Demographic and metabolic characteristics of RA patients

RA patients who underwent total knee arthroplasty in our hospital were collected (n = 29). According to the 2005 NCEP-ATP-III diagnostic criteria for Asian patients with MetS, RA patients with MetS were included in MetS-RA group (n = 15), and patients without MetS were included in nMetS-RA group (n = 14). All participants in the study obtained the consent of themselves or their families. The study was approved by the ethics committee of Henan Provincial People’s Hospital (IRB ID:2023-108). Demographics are shown in **Supplementary Table 1**. Serum metabolic indicators of RA patients were recorded.

### Human synovial sample collection and RA-FLS isolation

Synovium samples of RA patients were collected. The synovium was cut into 1 mm^3^ pieces, digested with 2.5 mg/ml type I collagenase at 37°C for 2 h, filtered with 75 μm filter to remove the tissue fragments. The isolated cells were cultured in DMEM medium containing 10% fetal bovine serum and 1% penicillin-streptomycin. RA-FLSs were passaged when reaching approximately 90% confluence. Macrophages were removed by passage to purify FLS. RA-FLSs of more than three passages were used for experiments.

### The validation of the expression of hub genes

Total cellular RNA of RA-FLS was extracted using RNA extraction reagent (Accurate) according to the manufacturer’s instructions. cDNA synthesis (Accurate) and quantitative real-time PCR (qRT-PCR, Accurate) were performed according to the manufacturer’s instructions. Data of hub genes were expressed as 2^-△△ct^, mRNA expression was calculated relative to the *β-actin* level. Primer sequences are listed as follows: *TYK2*-F, 5’-GAGATGCAAGCCTGATGCTAT-3’, *TYK2*-R, 5’-*GGTTCCCGAGGATTCATGCC*-3’, *TRAF2*-F, 5’-*TCCCTGGAGTTGCTACAGC*-3’, *TRAF2*-R, 5’-*AGGCGGAGCACAGGTACTT*-3’.

### The effect of small molecule compounds on cell function

The effects of small molecule compounds on the function of RA-FLS were evaluated by wound-healing assays and angiogenesis experiments.

### Cell counting kit-8 for cell viability

CCK-8 was used to determine the viability of RA-FLS incubated with camptothecin (CPT, TargetMol) in a concentration gradient (0, 2.5, 5, 10, 20 µmol/L) and a time gradient (6, 12, 24, 48, 72 h). Cell viability (%) = [OD value (CPT) - OD value (blank)]/[OD value (control) - OD value (blank)]×100%. According to CCK-8 results, optimal concentration of CPT was determined to be 5 µmol/L.

### Wound-healing assays

2×10^5^ RA-FLSs/well were seeded in 6-well plates, and were grown overnight until they reached 95% confluence, after which wound gaps were generated using a 200µl sterile pipette tip, and cellular debris was removed with phosphate buffered saline (PBS). The wound closure was monitored and photographed by inverted microscope (Olympus) at 0 h, 24 h, and 48 h. The migration area was quantified using imageJ (version 1.8.0) (21), average scratch width = (initial scratch area - scratch area at detection time)/image length, and cell migration ratio = (initial scratch area - scratch area at detection time)/initial scratch area.

### Angiogenesis experiments

Growth factor reduced matrigel (no phenol red, 8.9 mg/ml, Becton), 48-well plate and pipette tips were precooled at 4°C, 120 µl matrigel was uniformly added to 48-well plate and solidified at 37°C for 30 min. Human umbilical vein endothelial cells (HUVECs) were re-suspended with pre-collected serum-free culture supernatants (nMetS-RA-FLS, MetS-RA-FLS, and MetS-RA-FLS treated with 5 µmol/L CPT for 24 h), and 200 µl cell culture supernatant containing 8×10^4^ HUVEC was added to each well. HUVECs were cultured in a 5% CO_2_ cell incubator at 37°C for 4 h. The tube formation of HUVECs was observed with an inverted microscope and photographed. The number of meshes and segments length were quantized by imageJ (22).

### Statistical analysis

All data between the two groups were analyzed statistically and plotted using GraphPad Prism software (version 8.0) or Sangerbox Tools. The normality of the data distribution was evaluated using the Kolmogorov–Smirnov test. Levene test was used to assess the homogeneity of variance. Independent samples t-tests were applied to analyze normally distributed values. Data with non-Gaussian distribution were analyzed by using the non-parametric Mann–Whitney U test. Correlation analysis was performed using Spearman’s method. Two-tailed values of *P* < 0.05 were considered statistically significant.

## Results

### Data processing, identification of DEGs and GSEA enrichment analysis

**Figure 1:**
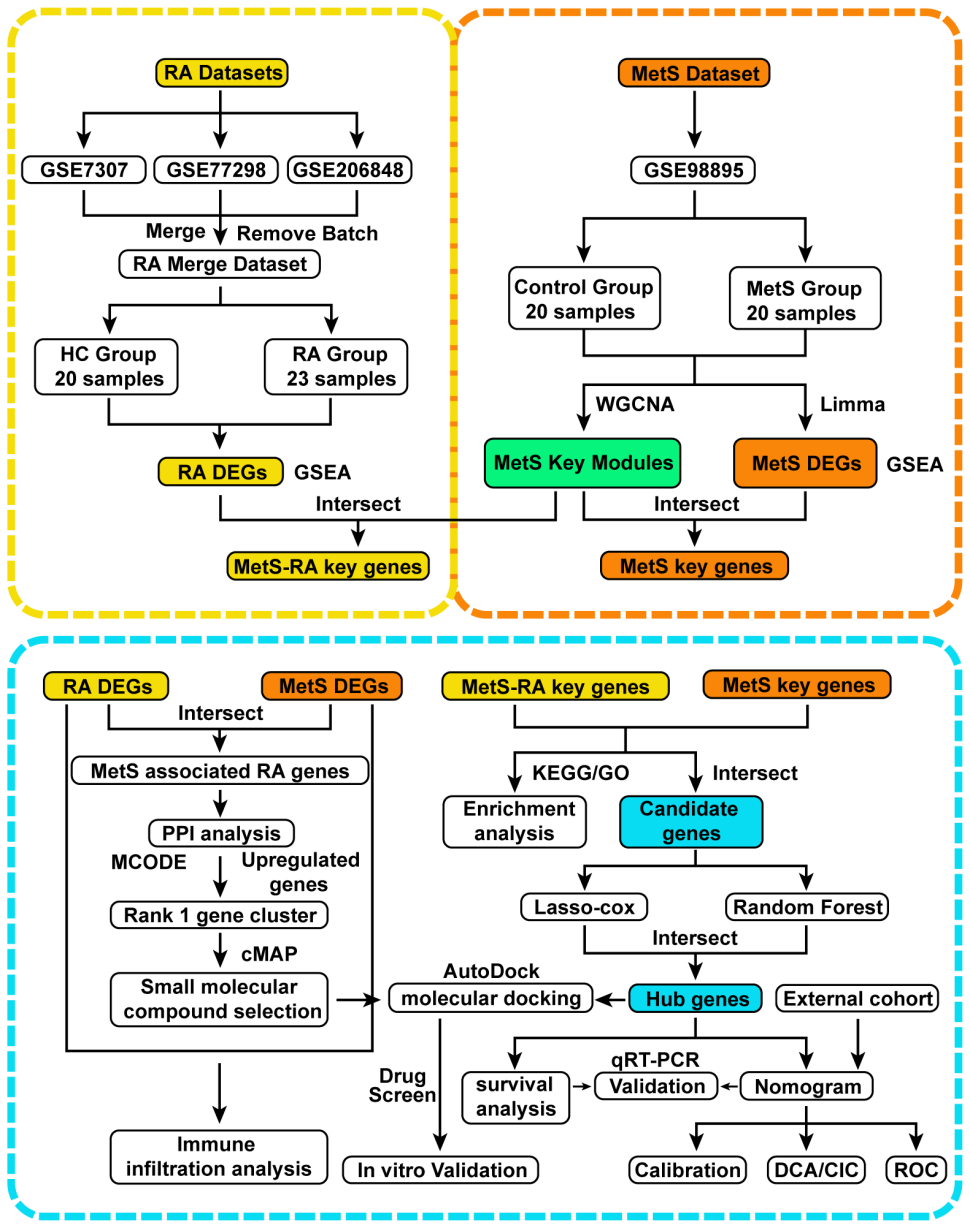
Flow chart of this study design. RA, Rheumatoid arthritis; MetS, Metabolic syndrome; MetS-RA, MetS-associated RA; DEGs, differentially expressed genes; WGCNA, weighted correlation network analysis; Gene Set Enrichment Analysis, GSEA; Limma, linear models for microarray data; PPI, protein-protein interaction; MCODE, molecular complex detection; KEGG, Kyoto Encyclopedia of Genes and Genomes; GO, Gene Ontology; least absolute shrinkage and selection operator, LASSO; qRT-PCR, quantitative real-time PCR; DCA, decision curve analysis; CIC clinical impact curve; ROC, receiver operating characteristic.

**Figure 2:**
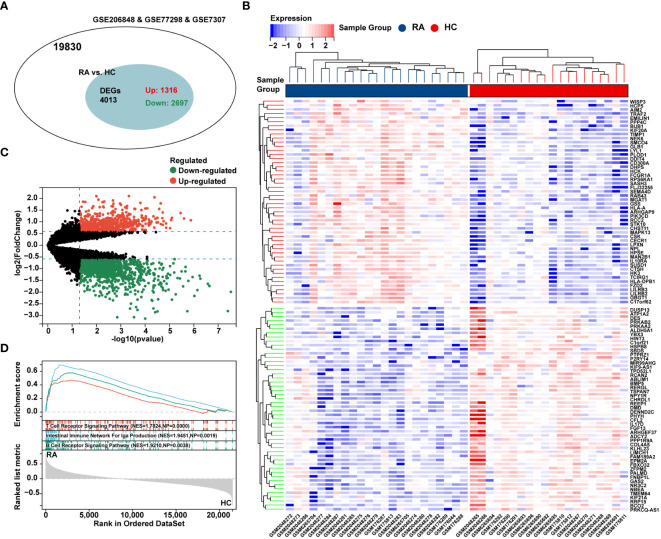
Differential expression analysis and GSEA of the integrated RA dataset. **(A)** The venn chart represented the total number of genes and the number of DEGs in the integrated RA dataset, with red representing up-regulated genes and green representing down-regulated genes. **(B)** The heatmap represented expression patterns of partial significantly up-regulated or down-regulated DEGs in the integrated RA dataset, with each row representing a DEG and each column representing a sample of RA cases or controls. **(C)** The volcano plots represented the expression pattern of RA DGEs with red representing up-regulated genes, green representing down-regulated genes, and black representing genes with no significant differences. **(D)** GSEA with KEGG gene sets indicated up-regulated T cell receptor signaling pathways, B cell receptor signaling pathways, and NK cell mediated cytotoxicity signaling pathways in RA synovium compared with healthy control (NES absolute value > 1.5, NP value < 0.05, FDR < 0.05). NES, normalized enrichment score; NP, Nominal *P* value; FDR, false discovery rate.

**Figure 3:**
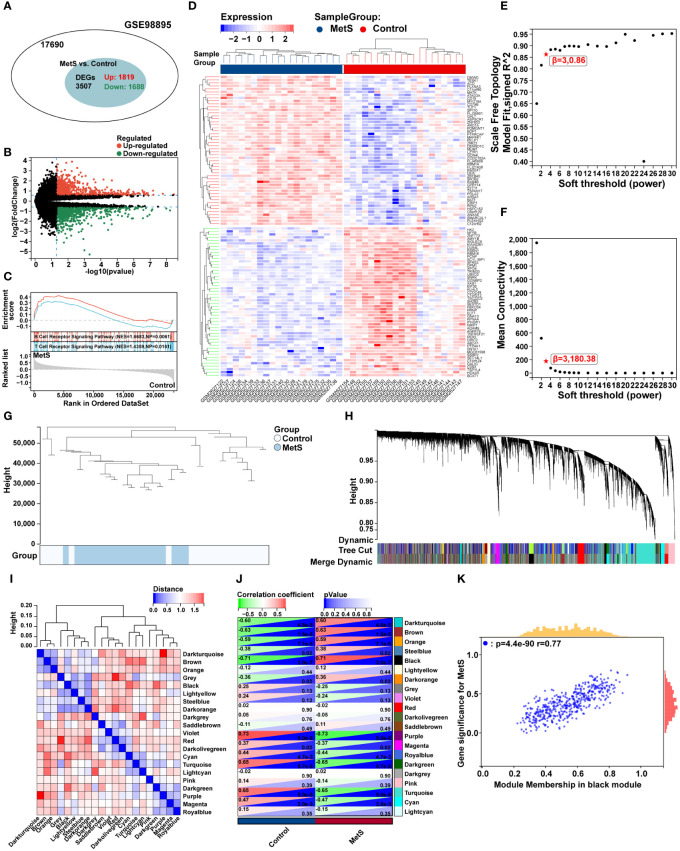
Differential expression analysis, GSEA analysis, and identification of key modules of WGCNA in the MetS dataset. **(A)** The venn chart represented the total number of genes and the number of DEGs in the MetS dataset, with red representing up-regulated genes and green representing down-regulated genes. **(B)** The volcano plots represented the expression pattern of MetS DGEs with red representing up-regulated genes, green representing down-regulated genes, and black representing genes with no significant differences. **(C)** GSEA with KEGG gene sets indicated up-regulated T cell receptor signaling pathways and B cell receptor signaling pathways in MetS PBMCs compared with healthy control (NES absolute value > 1.5, NP value < 0.05, FDR < 0.05). **(D)** The heatmap represented expression patterns of partial significantly up-regulated or down-regulated DEGs in the MetS dataset, with each row representing a DEG and each column representing a sample of MetS cases or controls. **(E)** A scale-free topological model was used to determine the optimal β value, combined with scale independence and **(F)** mean connectivity analysis, β = 3 was selected as the soft threshold. **(G)** The cluster dendrogram of the MetS and control samples. **(H)** Gene co-expression modules represented by different colors under the gene tree. **(I)** Heatmap of eigengene adjacency. **(J)** The heatmap represented the relationship between module eigengenes and MetS. The correlation (left) and *P* value (right) were presented. The black module correlated with RA exhibited the highest correlation coefficient, which was identified as the key module of MetS. **(K)** Correlation plot between module membership and gene significance of genes included in the black module. PBMCs, peripheral blood mononuclear cells.

The flow chart of bioinformatics analysis is shown in **Figure 1**. After batch correction, a total of 23 RA synovial samples and 20 healthy control synovium samples were obtained from the integrated RA dataset. All three datasets are from the same sequencing platform and contain the same number of genes (**Supplementary Figure 1A**). After batch effect removal, the data distribution among the three datasets tended to be consistent (**Supplementary Figures 1B–E**), and they cluster and intertwine with each other (**Supplementary Figures 1F, G**), indicating that the batch effect was better removed. There were 4013 DEGs in the integrated RA dataset, including 1316 up-regulated genes and 2697 down-regulated genes (**Figure 2A**). The volcano plot and heatmap were used to display the expression patterns of DEGs in the merged RA dataset (**Figures 2B, C**). GSEA analysis revealed up-regulated T cell receptor signaling pathways, B cell receptor signaling pathways, and Intestinal Immune Network For Iga Production (**Figure 2D**). To comprehensively demonstrate the metabolic characteristics of RA datasets, KEGG enrichment analysis involving metabolic pathway was conducted via GSEA. The results showed that these RA datasets have similar metabolic characteristics, including abnormalities in metabolic pathways such as Type I Diabetes Mellitus, Sulfur Metabolism, and Tyrosine Metabolism (**Supplementary Tables 2–4**). A total of 3507 DEGs were identified in MetS dataset, including 1819 up-regulated genes and 1688 down-regulated genes (**Figure 3A**). The volcano plot and heatmap were used to describe the expression patterns of DEGs in the MetS dataset (**Figures 3B, D**). GSEA analysis showed up-regulated T cell receptor signaling pathways and B cell receptor signaling pathways (**Figure 3C**). KEGG enrichment analysis involving metabolic pathway demonstrated the metabolic characteristics of MetS and dysregulated metabolic genes (**Supplementary Table 5**).

### Construction of WGCNA and identification of key module genes in MetS dataset

To further explore key genes in MetS, WGCNA was conducted to identify the most relevant gene modules in MetS dataset. Based on scale independence and mean connectivity, the soft threshold of soft power was set as 3 (**Figures 3E, F**). The clustering tree diagram of MetS and control group were shown in **Figure 3G**. In order to further assess these modules, we calculated the dissimilarity of module eigengenes, chose a cut line for module dendrogram and merged the module with a distance less than 0.25, and a total of 21 co-expression modules were obtained (**Figure 3H**). Spearman’s correlation coefficient was performed to map the module-trait relationship and evaluate the correlation between each module and MetS diagnosis (**Figure 3I**). The black module exhibited the highest positive correlation with MetS (454 genes, r = 0.71, *P* = 3e-7), and was selected as the most relevant module for MetS (**Figure 3J**). The correlation analysis results of genes in the black module showed a strong association between module membership and gene significance, r = 0.77, *P* = 4.4e-90 (**Figure 3K**).

### MetS key genes screening and enrichment analysis

**Figure 4:**
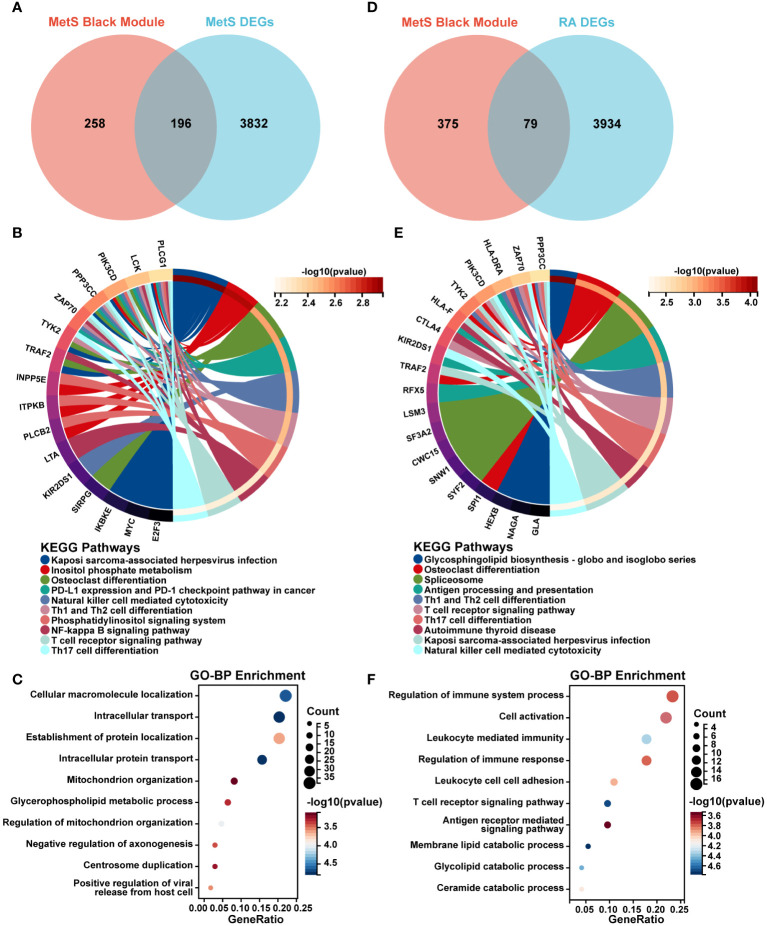
Screening and enrichment analysis of MetS key genes and MetS-RA key genes. **(A)** The venn diagram represented the overlap between MetS black module genes and MetS DEGs, totally 196 overlapping genes, which were defined as MetS key genes. Red represents MetS black module genes and blue represents MetS DEGs. **(B)** The circos plot represented KEGG analysis results for MetS key genes. **(C)** The bubble plot represented GO-BP analysis results for MetS key genes. **(D)** The venn diagram represented the overlap between MetS black module genes and RA DEGs, totally 79 overlapping genes, which were defined as MetS-RA key genes. Red represents MetS black module genes and blue represents RA DEGs. **(E)** The circos plot represented KEGG analysis results for MetS-RA key genes. **(F)** The bubble plot represented GO-BP analysis results for MetS-RA key genes. GO-BP, Gene Ontology Biological Process.

A total of 196 overlapping genes between MetS black module genes and MetS DEGs were identified (**Figure 4A**). KEGG enrichment analysis showed that these overlapping genes were mainly enriched in “Kaposi sarcoma-associated herpesvirus infection”, “osteoclast differentiation”, “NF-kappa B signaling pathway”, and immune cell-related signaling pathways such as “NK cell mediated cytotoxicity”, “Th1 and Th2 cell differentiation”, “Th17 cell differentiation”, and “T cell receptor signaling pathway” (**Figure 4B**). In terms of GO-BP analysis, overlapping genes were mainly enriched in “Cellular macromolecule localization”, “Intracellular transport”, “Intracellular protein transport”, “Regulation of mitochondrion organization” and other processes (**Figure 4C**).

### MetS-RA key genes screening and enrichment analysis

There were a total of 79 overlapping genes between MetS black module genes and RA DEGs (**Figure 4D**). KEGG enrichment analysis showed that these overlapping genes were concentrated in “Osteoclast differentiation”, “Spliceosome”, and immune cell-related signaling pathways such as “Th1 and Th2 cell differentiation”, “Th17 cell differentiation”, “NK cell mediated cytotoxicity”, and “T cell receptor signaling” (**Figure 4E**). In terms of GO-BP analysis, overlapping genes were mainly enriched in immune-related processes such as “Regulation of immune response”, “Cell activation”, “Leukocyte mediated immunity”, “Regulation of immune system process”, and “T cell receptor signaling pathway”. In addition, enrichment pathways were involved in metabolism-related pathway including “Membrane lipid catabolic process”, “Glycolipid catabolic process”, and “Ceramide catabolic process” (**Figure 4F**).

### Screening of hub genes for MetS-RA diagnosis via machine learning

**Figure 5:**
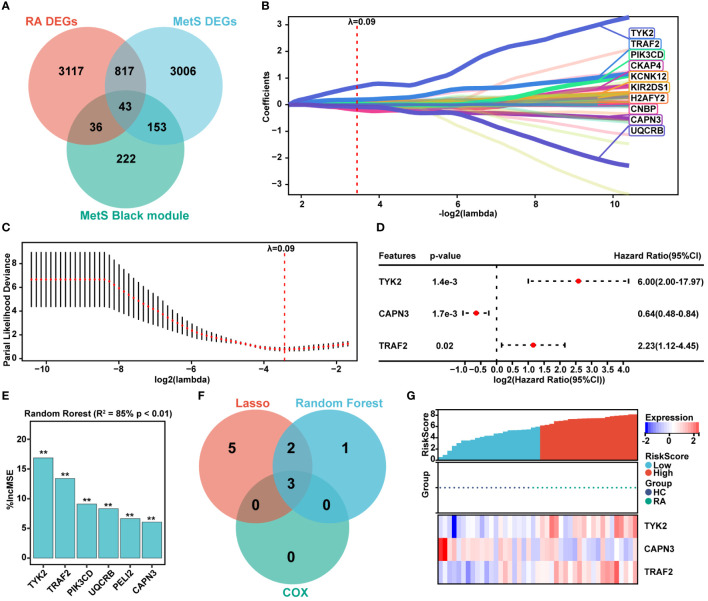
Hub genes screening via LASSO-COX and Random Forest. **(A)** The venn diagram represented the overlap among RA DEGs, MetS DEGs, and MetS black module genes, with a total of 43 overlapping genes defined as MetS-RA pathogenic candidate genes. Red represents RA DEGs, blue represents MetS DEGs, and green represents MetS black module genes. **(B, C)** The expression profile data of 43 genes were analyzed through the LASSO algorithm of Sangerbox Tools based on R software “glmnet” package. The R software “survival” package was used to integrate RA diagnosis and gene expression profile data, and the prognostic significance of each gene was further evaluated by COX method. The risk score was computed by the mRNA expression of diagnostic biomarkers weighted by their corresponding coefficients via Sangerbox Tools. The diagnostic biomarkers (n = 10) were identified by the LASSO logistic regression algorithm, λ = 0.09. **(D)** The forest plot represented candidate genes screened by LASSO model were evaluated for RA diagnostic significance by COX method. **(E)** The R software “random forest” package was also used to screen out potential hub genes. The diagnostic biomarkers (n = 6) were identified by the RF algorithm with %IncMSE *P* value < 0.01 were extracted. ** *P* < 0.01. **(F)** The venn diagram displayed three common genes between LASSO-COX and RF algorithms, which were identified as the hub genes in MetS-RA. **(G)** Visualization of the relationship between RA diagnostic risk and hub genes expression.

**Figure 6:**
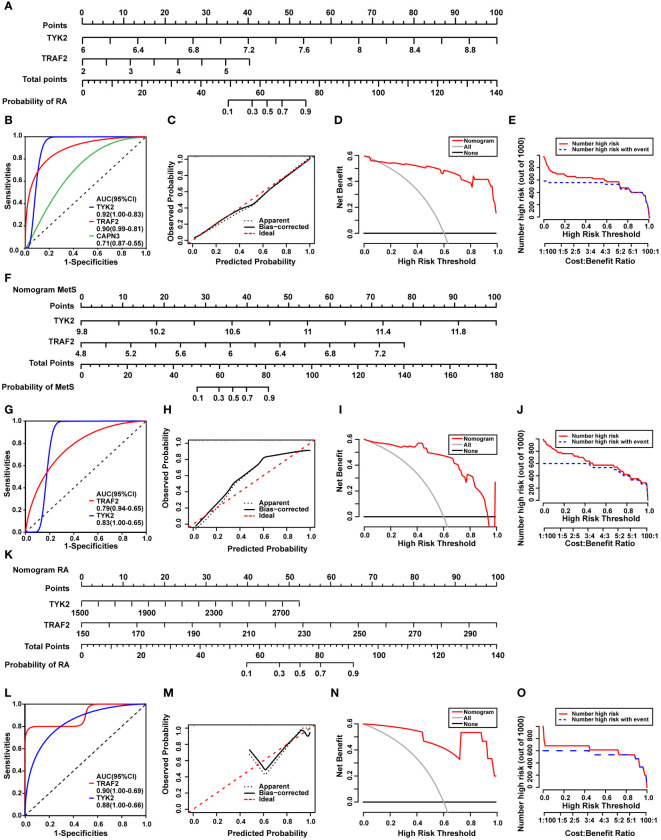
Construction of a diagnostic nomogram model and evaluation of diagnostic models in external cohorts. **(A)** The nomogram was constructed based on the hub genes *TYK2* and *TRAF2*. **(B)** The ROC curve for the diagnostic performance of *TYK2*, *TRAF2*, and *CAPN3*. **(C)** The calibration curve of nomogram model prediction in MetS-RA. The dotted line marked as “Apparent” represents the uncalibrated prediction curve. The solid line marked as “Bias−corrected” represents the calibrated prediction curve. The dash line marked as “Ideal”, represents the perfect prediction of the ideal model. **(D)** DCA for the nomogram model. The black line marked as “None” stands for the net benefit of the assumption that no patients have RA. The grey line marked as “All” represents the net benefit of the assumption that all patients have RA. The red line marked as “Nomogram” represents the net benefit of the assumption that MetS-RA was identified according to the diagnostic value of RA predicted by the nomogram model. **(E)** CIC for the nomogram model. The red curve indicates the number of people who are classified as high risk by the model at each threshold probability. The blue curve represents the number of true positives at each threshold probability. **(F)** The nomogram was developed based on *TYK2* and *TRAF2* from MetS dataset (GSE142401) to predict the risk of MetS. **(G)** The ROC curves for the predictive performance of *TYK2*, *TRAF2*. **(H)** The calibration curve of nomogram prediction in MetS patients. **(I)** DCA for the nomogram model. **(J)** CIC for the nomogram model. **(K)** The nomogram was developed based on *TYK2* and *TRAF2* from RA dataset (GSE97779) to predict the risk of RA. **(L)** The ROC curves for the predictive performance of *TYK2*, *TRAF2*. **(M)** The calibration curve of nomogram prediction in RA patients. **(N)** DCA for the nomogram model. **(O)** CIC for the nomogram model.

The common DEGs between MetS and RA may play critical roles in MetS-RA, so the next step will be to further search for hub genes in MetS-RA. A total of 43 overlapping genes between MetS key genes and MetS-RA key genes will be further screened as candidate genes (**Figure 5A**). LASSO regression algorithm was applied to identify 10 potential candidate genes from 43 common genes (**Figures 5B, C**). The prognostic significance of each gene was further evaluated by COX regression analysis, and *TYK2*, *TRAF2* and *CAPN3* were screened out. The hazard ratio (HR) of *TYK2* was 6.00 (95%CI 2.00 - 17.97), the HR of *TRAF2* was 2.23 (95%CI 1.12 - 4.45), and the HR of *CAPN3* was 0.64 (95%CI 0.48 - 0.84) (**Figure 5D**). Meanwhile, RF machine learning algorithm was applied to sort 43 overlapping genes according to the %IncMSE of each gene, and genes with %IncMSE *P* value < 0.01 were extracted. A total of 6 genes were obtained (**Figure 5E**). Three overlapping genes (*TYK2*, *TRAF2*, and *CAPN3*) were identified in candidate genes obtained by LASSO-COX and RF (**Figure 5F**). The expression of *TYK2* and *TRAF2* was positively correlated with the risk score of RA diagnosis, while *CAPN3* was negatively correlated with the risk score of RA diagnosis (**Figure 5G**). For better diagnosis and prediction, a nomogram was constructed based on *TYK2* and *TRAF2* by logistic regression analysis (**Figure 6A**). The area under the curve (AUC) value of the hub gene was evaluated using ROC to determine its sensitivity and specificity for the diagnostic efficacy of MetS-RA. AUC values of *TYK2* and *TRAF2* were both > 0.9, suggesting that these two hub genes had strong diagnostic value for MetS-RA (**Figure 6B**). The calibration curve showed that the prediction probability of the constructed nomogram diagnostic model was almost identical to that of the ideal model (**Figure 6C**). In addition, the DCA and the CIC showed that decision making based on the nomogram model may be beneficial for the diagnosis of MetS-RA (**Figures 6D, E**). To further confirm the accuracy of the above nomogram model, two external GEO datasets were used for validation. We developed a MetS diagnostic nomogram model based on GSE142401 to predict the possibility of MetS from control and MetS groups (**Figure 6F**). The AUC values of *TYK2* and *TRAF2* were 0.83 and 0.79, respectively (**Figure 6G**). The calibration curves, DCA, and CIC for assessing nomogram MetS showed that decision-making based on the nomogram MetS may favor the prediction of MetS (**Figures 6H–J**). Furthermore, another diagnostic nomogram model was also constructed to distinguish RA patients based on GSE97779 (**Figure 6K**). Similarly, ROC, calibration curves, DCA, and CIC indicated ideal predictive value of nomogram RA for the RA patients (**Figures 6L–O**).

### Identification of candidate small molecule compounds and molecular docking

**Figure 7:**
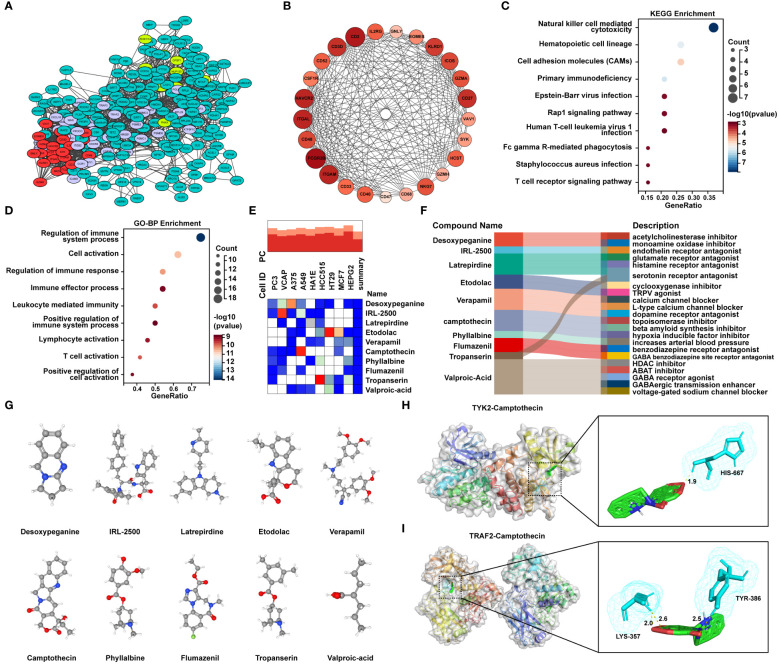
Identification of candidate small molecule compounds and molecular docking. **(A)** PPI analysis of overlapping genes between RA DGEs and MetS DEGs was performed by STRING database, and cluster analysis was performed via MCODE function of Cytoscape software. **(B)** The PPI network of module genes with the highest score contained 25 genes based on MCODE analysis. **(C)** The bubble plot displayed the KEGG enrichment analysis and **(D)** GO-BP enrichment analysis of genes included in module with the highest score. **(E)** The heatmap presented the top10 compounds with the most significantly negative enrichment scores in cell lines based on CMap analysis. **(F)** The sankey diagram showed a description of the top ten compounds. **(G)** 3D chemical structure visualization of 10 compounds. **(H)** The molecular docking of TYK2 and camptothecin (CPT), and **(I)** the molecular docking of TRAF2 and CPT. The yellow dash line represents the hydrogen bond between CPT and the amino acid residue of TYK2 or TRAF2.

In order to uncover potential pathogenic genes and underlying signaling pathways in MetS-RA, interactions of MetS associated RA genes were obtained through the STRING database with a medium confidence score > 0.4. The top-ranked module was identified and visualized via the MCODE function of Cytoscape software (**Figure 7A**, **Supplementary Figures 2A**). The module with the highest score contained 25 genes (**Figure 7B**). KEGG analysis showed that these genes were mainly enriched in “NK cell mediated cytotoxicity”, “Hematopoietic cell lineage”, “Cell adhesion molecules”, and immune-related signaling pathways (**Figure 7C**). In terms of GO-BP, overlapping genes were mainly enriched in immune regulatory processes such as “Regulation of immune system process”, “Regulation of immune response” and “Positive regulation of immune system process”, and cell activation processes such as “T cell activation”, “Lymphocyte activation”, and “Positive regulation of cell activation” (**Figure 7D**). To predict potential small molecule compounds that might play a therapeutic role in MetS-RA patients, 25 genes (all up-regulated genes) were analyzed via the CMap database. The top 10 compounds with the highest negative scores were considered to be potential therapeutic drugs for the treatment of MetS-RA, including desoxypeganine, IRL-2500, latrepirdine, etodolac, verapamil, CPT, phyllalbine, flumazenil, tropanserin, and valproic-acid (**Figure 7E**). The sankey diagram showed the targeted pathways of these 10 compounds (**Figure 7F**), and the chemical structure was visualized (**Figure 7G**). Further, the molecular docking of these potential therapeutic compounds with TYK2 and TRAF2 was carried out, and the results showed that IRL-2500 and CPT were the top two compounds most closely bound to TYK2 or TRAF2. The binding energy of TYK2 with IRL-2500 was -10.3 kcal/mol, and the binding energy of TYK2 with CPT was -9.43 kcal/mol. TRAF2 has a binding energy of -8.24 kcal/mol with IRL-2500 and -7.7 kcal/mol with CPT (**Supplementary Figures 2B, C**). It was well proved from a previous research that low-dose CPT could improve the disease condition of RA (23), so the next step was to validate the effect of CPT on the function of MetS-RA-FLS *in vitro*. The molecular docking of TYK2 or TRAF2 with CPT was visualized (**Figures 7H, I**).

### Validation of the expression of hub genes and the effect of camptothecin on cell migration and angiogenesis of MetS-RA-FLS

**Figure 8:**
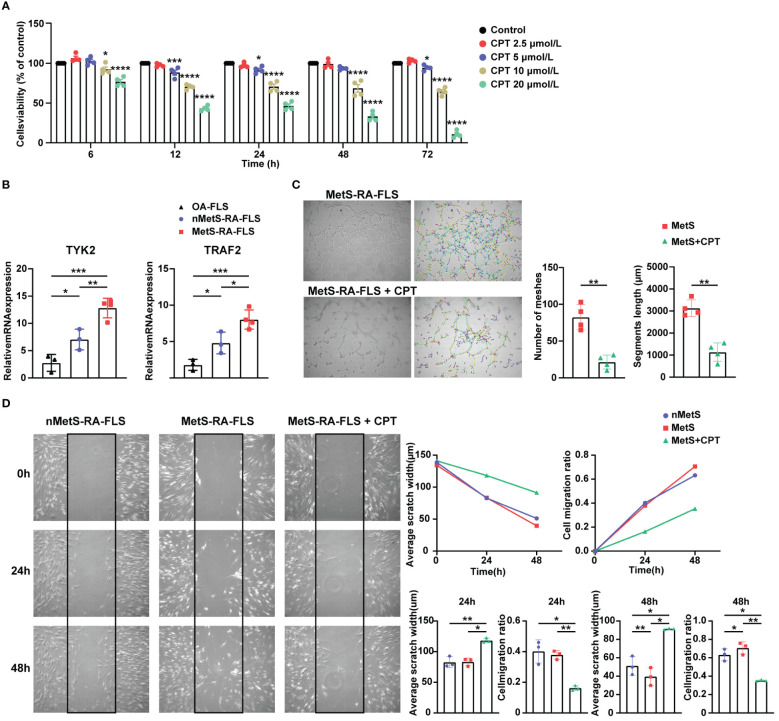
Validation of the expression of hub genes and the effect of CPT on cell migration and angiogenesis of MetS-RA-FLS. **(A)** Cell Counting Kit-8 (CCK-8) was used to determine the viability of RA-FLS incubated with CPT in a concentration gradient (0, 2.5, 5, 10, and 20μmol/L) and a time gradient (6, 12, 24, 48, and 72h). **(B)** qRT-PCR was used to determine the relative mRNA level of *TYK2* and *TRAF2* in OA-FLS (n = 3), nMetS-RA-FLS (n = 3), and MetS-RA-FLS (n = 4). Data are mean ± SD, * *P* < 0.05, ** *P* < 0.01, *** *P* < 0.001, **** *P* < 0.0001. **(C)** Angiogenesis experiments exhibited HUVEC tube formation after 4h treatment with cell culture supernatants of MetS-RA-FLS and MetS-RA-FLS incubated with 5 µmol/L CPT (n = 4, per group). Number of meshes and segments length were calculated via ImageJ. **(D)** Wound-healing assays displayed cell migration of nMetS-RA-FLS, MetS-RA-FLS, and MetS-RA-FLS treated with 5 µmol/L CPT at 0, 24, and 48 h (n = 3, per group). Measurement of scratch width and cell migration ratio via ImageJ.

To verify the accuracy of the two hub genes obtained by above integrated bioinformatics analysis, FLSs from patients with osteoarthritis (OA), MetS-RA, and nMetS-RA were isolated. CCK-8 assay showed that the cytotoxicity of CPT to RA-FLS was obvious with the concentration of CPT at 10 or 20μmol/L. According to CCK-8 results, optimal concentration of CPT was determined to be 5 µmol/L (**Figure 8A**). qRT-PCR results indicated that *TYK2* and *TRAF2* levels were elevated in RA-FLS compared to OA-FLS, *TYK2* and *TRAF2* were higher expressed in MetS-RA-FLS compared to nMetS-RA-FLS (**Figure 8B**). Then CPT was identified as a potential treatment for MetS-RA. RA-FLS has the ability to promote angiogenesis and strong cell migration, so wound-healing assays and angiogenesis experiments were conducted *in vitro* to verify the effect of CPT on MetS-RA-FLS functions. The results revealed that CPT significantly attenuated the angiogenesis ability of MetS-RA-FLS (**Figure 8C**). Wound-healing assays showed that MetS-RA-FLS had stronger cell migration ability than MetS-RA-FLS, and CPT treatment significantly reduced the cell migration ability of MetS-RA-FLS (**Figure 8D**).

### Immune cell infiltration and correlation analysis in MetS and RA

**Figure 9:**
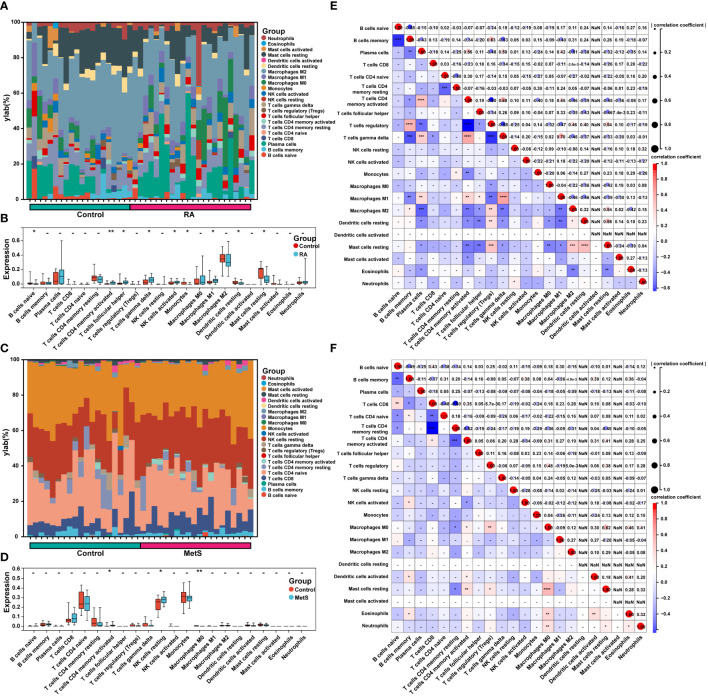
Immune cell infiltration and correlation analysis in MetS and RA. **(A)** The stacked histogram displayed the proportion of 22 kinds of immune cells between RA and control groups. **(B)** The boxplot showed the comparison of 22 kinds of immune cells between RA and control groups. Data are mean ± SD, * *P* < 0.05, ** *P* < 0.01. **(C)** The stacked histogram represented the proportion of 22 kinds of immune cells between MetS and control groups. **(D)** The boxplot exhibited the comparison of 22 kinds of immune cells between MetS and control groups. Data are mean ± SD, * *P* < 0.05, ** *P* < 0.01. **(E)** The heatmap displayed the correlation of 22 immune cell type compositions. Both horizontal and vertical axes demonstrate immune cell subtypes of RA or **(F)** MetS. * *P* < 0.05, ** *P* < 0.01, *** *P* < 0.001, **** *P* < 0.0001.

The enrichment analysis of MetS key genes and MetS-RA key genes showed that MetS and MetS-RA were closely related to immune cell-related signaling pathways. Therefore, the CIBERSORT algorithm was employed to obtain immune cell characteristics to explore the immune regulation and the correlation between infiltrating immune cells in MetS and RA. Immunoinfiltration analysis displayed the proportion of 22 types of immune cells in each sample (**Figures 9A, C**). There were significant differences in 9 immune cell subsets between RA and control synovium samples. Compared with the control group, the proportion of T cells CD4 memory activated, T cells follicular helper, T cells gamma delta, NK cells activated, and Macrophages M1 increased, while the proportion of B cells naive, Monocytes, Dendritic cells resting, and Mast cells resting decreased (**Figure 9B**). The correlation analysis showed that Macrophages M1 was significantly positively correlated with T cells gamma delta (r = 0.70, *P* < 0.0001), T cells CD4 memory activation was negatively correlated with T cells regulation (r = -0.60, *P* < 0.0001) (**Figure 9E**). There were significant differences between the PBMC samples from MetS and control groups in 3 immune cell subsets, and the proportion of T cells CD4 memory activated and NK cells resting was increased in MetS PBMC, while the proportion of Macrophages M0 decreased (**Figure 9D**). The correlation analysis showed that Macrophages M0 was significantly positively correlated with Mast cells resting (r = 0.62, *P* < 0.0001), T cells CD8 was negatively correlated with T cells CD4 memory resting (r = -0.60, *P* < 0.0001) (**Figure 9F**).

## Discussion

In recent years, with the widespread application of sequencing, integrated bioinformatic analysis and machine learning methods are increasingly being applied to explore key genes of disease, potential diagnostic and prognostic biomarkers, potential signaling pathways, and therapeutic targets, thereby providing data support for the full disclosure of diseases (24, 25). The association between RA and MetS has received extensive attention, and a large number of studies have revealed a close relationship between various components of MetS and clinical features of RA (8, 26). In terms of molecular mechanisms, adipokines associated with MetS and metabolic reprogramming of cells caused by MetS affect the disease course of RA (4). However, studies on RA and MetS based on bioinformatics are much less explored. In this study, a variety of bioinformatics analysis methods and machine learning methods were employed to excavate pathogenic genes in MetS-RA, clarify the association between RA and MetS, predict small molecule compounds with potential therapeutic effects, and reveal the immune cell infiltration characteristics of MetS and RA, providing new ideas for future treatment of MetS-RA patients.

Firstly, the enrichment analysis in this study demonstrated that the pathogenesis of MetS-RA may focus on immune system regulation and immune cell activation. GSEA analysis revealed that both RA and MetS had up-regulated T cell receptor pathways and B cell receptor signaling pathways. Moreover, the key genes of MetS and MetS-RA that overlapped with key modules of WGCNA were enriched in Th1/2/17 cell differentiation, NK mediated cytotoxicity, and osteoclast differentiation signaling pathways, which were also included in the enrichment pathway of MetS and RA overlapping DEGs. Li et al. have enlightened the contribution of NK cells to the chronic inflammatory state found in obesity (27). NK cells are innate lymphoid cells that reside in visceral adipose tissue and mediate cellular cytotoxicity. In addition, a study has demonstrated that in non-obese people, macrophages were the main immune cells in adipose tissue, playing a key role in regulating anti-inflammatory mediators, while in obese people, the shift in macrophage phenotype leads to the recruitment of NK cells (28). In turn, NK cells produce cytokines and chemokines that further promote the inflammatory microenvironment associated with obesity. Th cells are key regulators of pro-inflammatory and anti-inflammatory immune processes. Th1 cells are pro-inflammatory cells that express the transcription factor T-bet, interferon γ (IFNγ), interleukin 2 (IL-2), and tumor necrosis factor α (TNF-α). Th17 cells are also highly pro-inflammatory cells. While in obesity-related conditions, Th2 cells seem to exert an anti-inflammatory effect. At present, it is well confirmed that Th1 cells are involved in adipose tissue inflammation associated with obesity-related pathology, the content of Th17 cells in adipose tissue and peripheral blood was increased in patients with obesity and type 2 diabetes. In addition, Th1 and Th17 also aggravate insulin resistance (29). Yokota et al. have reported that osteoclasts induced by TNF and IL-6 participate in the pathological process of RA joint destruction (30). Moreover, pro-inflammatory systemic condition and altered immune response in MetS also affect both catabolic and anabolic processes of bone healing, including increased osteoclastogenesis and impaired osteoblast activity, which could be explained by the dysfunction of insulin receptors that led to activation of signals related to osteoblast differentiation (31). Furthermore, dysregulated metabolic genes existed in MetS, primarily involving glycan biosynthesis, inositol phosphate metabolism, and tryptophan metabolism. These abnormal metabolic pathway were also present in RA, indicating that MetS and RA may share common metabolic pathways, which may be a potential pathogenic mechanism for MetS-RA. In summary, abnormal immune cell differentiation and osteoclast differentiation in MetS may aggravate the pathological process of RA, and the immune environment of RA may also aggravate the inflammatory microenvironment and metabolic reprogramming of histocytes in adipose tissue of individuals with MetS.

Next, two hub genes of MetS-RA, *TYK2*, and *TRAF2*, were screened by machine learning methods. After the evaluation of ROC, DCA, and CIC, a nomogram model was constructed based on *TYK2* and *TRAF2* to show the ideal efficacy of RA diagnosis. In addition, qRT-PCR results showed higher levels of *TYK2* and *TRAF2* in MetS-RA-FLS, providing a potential novel serum biomarker for the diagnosis of MetS-RA. The tyrosine kinase encoded by the *TYK2* gene, a member of the Janus kinase (JAK) protein family, binds to cytoplasmic domains of type I and type II cytokine receptors and transmits cytokine signals by phosphorylating the receptor subunits. Currently, five different JAK inhibitors have been marketed as molecular-targeted compounds for RA as one of the therapeutic strategies for RA, and four of them inhibit *TYK2* (32). A Mendelian randomization study suggested that *TYK2* gene expression was closely associated with RA, and *TYK2* inhibition was associated with a reduced risk of multiple autoimmune diseases (33). A recent review suggested that MetS should be a new indication for JAK inhibitors, highlighting the potential role of JAK inhibitors in reducing relevant inflammatory processes, improving insulin sensitivity, and resolving crosstalk with insulin pathways (34). The protein encoded by *TRAF2* is a member of the TNF receptor-associated factor protein family. TRAF proteins are associated with members of the TNF receptor superfamily and mediate relevant signal transduction. Surveys such as that conducted by Potter et al. have shown that TRAF2 activated the NF-κB and JNK signaling pathways, which were involved in cell proliferation, cell differentiation, apoptosis, and bone remodeling. Importantly, it can further activate a variety of inflammatory and immune-related processes induced by cytokines such as TNF-α and IL-1, which play an important role in the pathology of RA (35). Likewise, Wu et al. found that in synovium tissues and cells of RA patients, TRAF2 methylation promoted sustained sensitization of NF-κB signal transduction by inhibiting its proteolysis and enhancing its activity, thereby promoting and sustaining inflammation in the joint (36). Interestingly, fisetin, a natural flavonoid drug that can alleviate MetS, eliminates high-fat diet-induced cardiac tissue inflammation induced by MetS via inhibiting TNFR1/TRAF2 signaling (37), suggesting that TRAF2 may be a key target in the systemic inflammatory response caused by MetS. Previous studies have screened many diagnostic biomarkers for RA and MetS through integrated bioinformatics analysis. Zhang et al. found that *SLAMF8* can be used as diagnostic biomarkers for RA (38), while Yu et al. confirmed that *LSP1* and *GNLY* have high predictability for RA (39). In the study by Li et al., *FZD7*, *IRAK3*, *KDELR3*, *PHC2*, *RHOB*, *RNF170*, *SOX13*, and *ZKSCAN4* were screened as hub genes between OA and MetS (25). These hub genes provide new evidence for future disease diagnosis of RA and MetS, and our results also enrich the spectrum of diagnostic biomarkers for RA and MetS.

Nowadays, three categories of disease-modifying anti-rheumatic drugs (DMARDs) constitute the therapeutic armamentarium of RA, including conventional synthetic DMARDs which are small-molecular-weight synthetic drugs with unclear anti-inflammatory mechanisms, biological DMARDs which are mostly monoclonal antibodies that specifically target an individual molecule, and targeted synthetic DMARDs which target specific enzymes within cells (1). However, the phenomenon of pathological metabolic changes caused by RA treatment drugs is of great concern. Previous studies have reported that rheumatoid cachexia can persist in RA patients receiving biotherapy, even after arthritis symptoms improve, and treatment with anti-TNF preparations and other biologic therapies may result in elevated lipid subcomponents. In addition, some medications can also improve insulin sensitivity and have different effects on adipokines (4). Thus, it is necessary to search for drugs that can both improve RA symptoms and metabolic abnormalities. Small molecule compounds exhibit several advantages, including high tissue penetration, a tunable half-life, and oral bioavailability, making them more effective (40). Our study identified a potential small molecule compound CPT targeting MetS-RA based on MetS-RA related pathogenic genes through CMap analysis, providing a new research perspective for the treatment of MetS-RA. CPT is a natural compound originally derived from the Asian camptotheca acuminata, synthesized by Wall and Wani in 1966. CPT is a topoisomerase I inhibitor originally used as an anti-cancer drug, blocks DNA synthesis and cell division leading to programmed cell death by inhibiting topoisomerase I-DNA complex (41). An *in vitro* study conducted by Jackson et al. proved that CPT could inhibit synovial cell proliferation, angiogenesis, and collagenase expression, with potential anti-arthritic effects, supporting the explorative use of topoisomerase I (especially CPT) and II inhibitors as potential agents against RA (23). And Koo et al. also performed a study *in vivo* demonstrated that subcutaneous injection of low-dose CPT could reduce joint inflammation in collagen-induced arthritis mice (42). In line with previous studies, we also confirmed that CPT significantly inhibited the cell migration and angiogenesis of MetS-RA-FLS *in vitro*.

There is a large number of published studies on the analysis of immune cell infiltration in RA synovium have shown that there was accumulation of various types of immune cells in RA synovium, including T cells, B cells, macrophages, NK cells, mast cells, and dendritic cells (39, 43, 44). CD4(+) T cells account for a large proportion of the immune cells invading the RA synovium and participate in the pathological process of RA. T follicular helper (Tfh) cell is a subtype of CD4(+) T cells, whose main functions are assisting B cells and regulating the production of antibodies. Tfh cell surface and secreted molecules, including CXCR5, ICOS, and PD1, are involved in the development of RA. Regulatory T (Treg) cells control an excess of T-cell-mediated immune responses, and Treg cell dysfunction can lead to the development of autoimmunity (45). CD8(+) T cells have anti-inflammatory properties that may help reduce ongoing autoimmune reactions in RA joints (46). NK cell-derived cytokines and their cytotoxic functions through induction of apoptosis take part in the regulation of the immune responses and could contribute to the pathogenesis of RA (47). Macrophages in the synovial membrane of RA are mainly differentiated into M1 type, which has a pro-inflammatory effect, and participate in the pathological process of RA by secreting cytokines to recruit mononuclear or neutrophilic granulocytes, activate T cells, and promote FLS proliferation and activation to promote inflammation (48). B cells in RA synovium induce the production of cytokines such as IL-1α, IL-23, IL-12, IL-6, and TNF-α, causing bone damage, inflammation, and immune disorders (49). Mast cells are tissue-resident cells of the innate immunity, they are present in synovium and their activation has been linked to the potentiation of inflammation in the course of RA (50). In addition, dendritic cells also have been shown to play an important role in the development of RA (51). In this study, immunoinfiltration analysis of RA synovium showed that CD4(+) T cells, T follicular helper cells, T gamma delta cells, NK cells, and M1 type macrophages increased, which was consistent with previous studies. In addition, our results showed that CD4(+) T cells and NK cells were higher in MetS PBMC than in controls, suggesting abnormal immune cell regulation in the peripheral blood of MetS patients. Therefore, a comprehensive understanding of immune cell infiltration associated with RA synovium is essential to explore novel diagnostic or prognostic biomarkers for RA and to further search for therapeutic targets.

Although bioinformatics is widely applied to screen disease diagnostic markers, however, due to individual differences in samples, diagnostic biomarkers may produce false positive or false negative results, leading to insufficient sensitivity and specificity of the biomarkers. Therefore, future research should set more standards to reduce the heterogeneity between samples and ensure the reliability of diagnostic biomarkers.

## Data availability statement

The datasets presented in this study can be found in online repositories. The names of the repository/repositories and accession number(s) can be found in the article/**Supplementary Material**.

## Ethics statement

The studies involving humans were approved by Ethics Committee of Henan Provincial People’s Hospital. The studies were conducted in accordance with the local legislation and institutional requirements. The participants provided their written informed consent to participate in this study.

## Author contributions

YH: Conceptualization, Data curation, Methodology, Software, Validation, Visualization, Writing – original draft, Writing – review & editing. SY: Data curation, Software, Validation, Writing – original draft. JQ: Data curation, Methodology, Visualization, Writing – review & editing. YD: Data curation, Writing – original draft. YL: Data curation, Writing – original draft. MZ: Software, Writing – original draft. CZ: Software, Writing – original draft. CC: Supervision, Writing – review & editing. YT: Conceptualization, Methodology, Software, Writing – review & editing. JZ: Conceptualization, Supervision, Writing – review & editing.
