# Supplementary material for: Identification of diagnostic genes and drug prediction in metabolic syndrome-associated rheumatoid arthritis by integrated bioinformatics analysis, machine learning, and molecular docking
Source: Front Immunol. 2024 Jul 29;15:1431452. doi: 10.3389/fimmu.2024.1431452 (PMC11320606; doi:10.3389/fimmu.2024.1431452)
Supplement: Supplementary file 1 [file DataSheet_1.docx]

**Identification of diagnostic genes and drug prediction in metabolic syndrome-associated rheumatoid arthritis by integrated bioinformatics analysis, machine learning, and molecular docking**

**
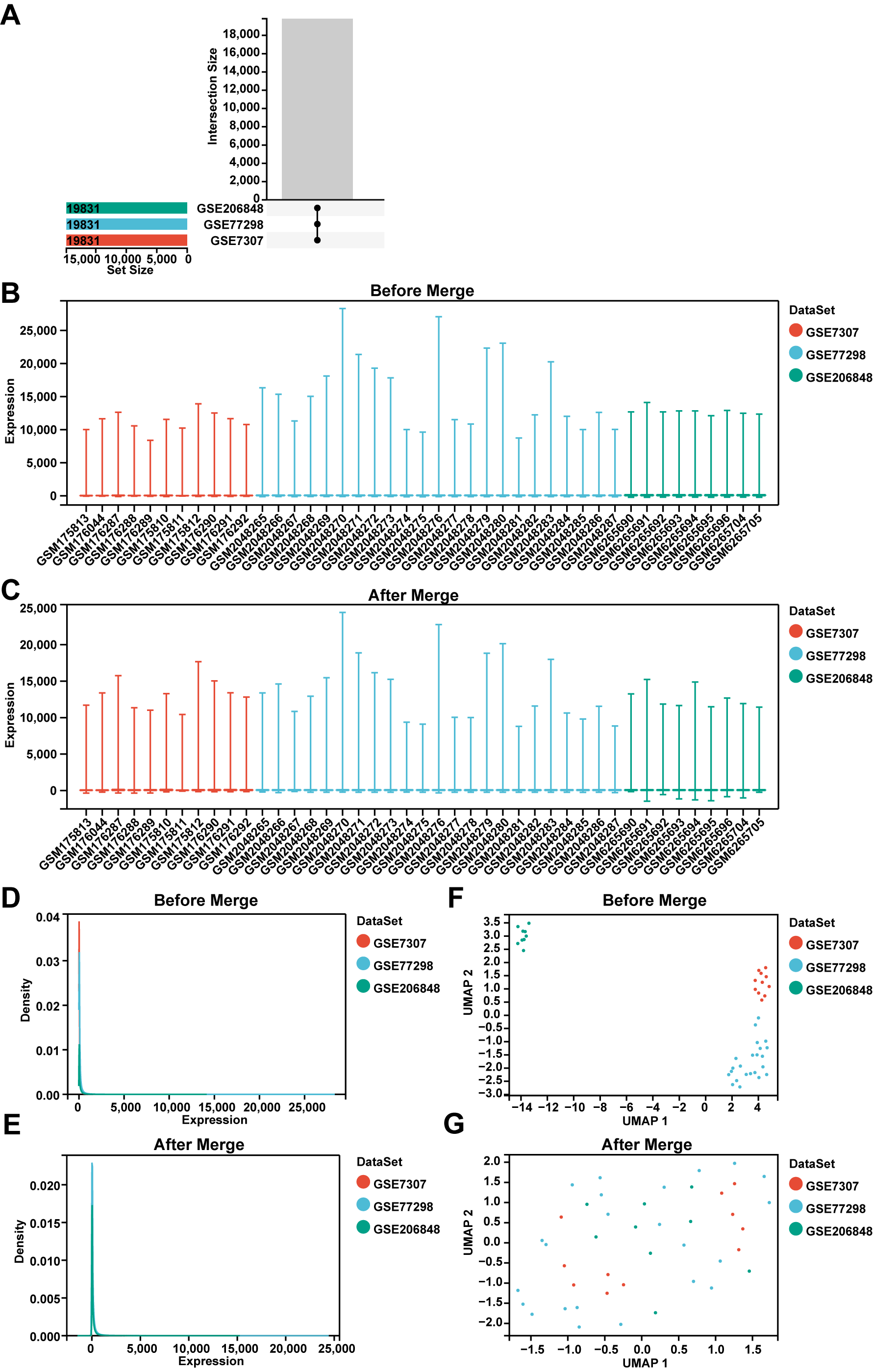
**

**Supplementary Figure 1**

The integration of RA datasets. (A) Three datasets (GSE206848, GSE77298, and GSE7307) are from the same sequencing platform and contain the same number of genes. (B) The box plot and (D) density plot represent the sample distribution of the individual datasets before the batch effect is removed. (C) The box plot and (E) density plot represent the sample distribution of each dataset after the batch effect is removed. (F) The UMAP plot showed that before the removal of batch effect, the samples of each dataset were clustered together, and (G) after the removal of batch effect, the samples of each dataset were clustered and intertwined with each other, indicating that the batch effect was better removed.


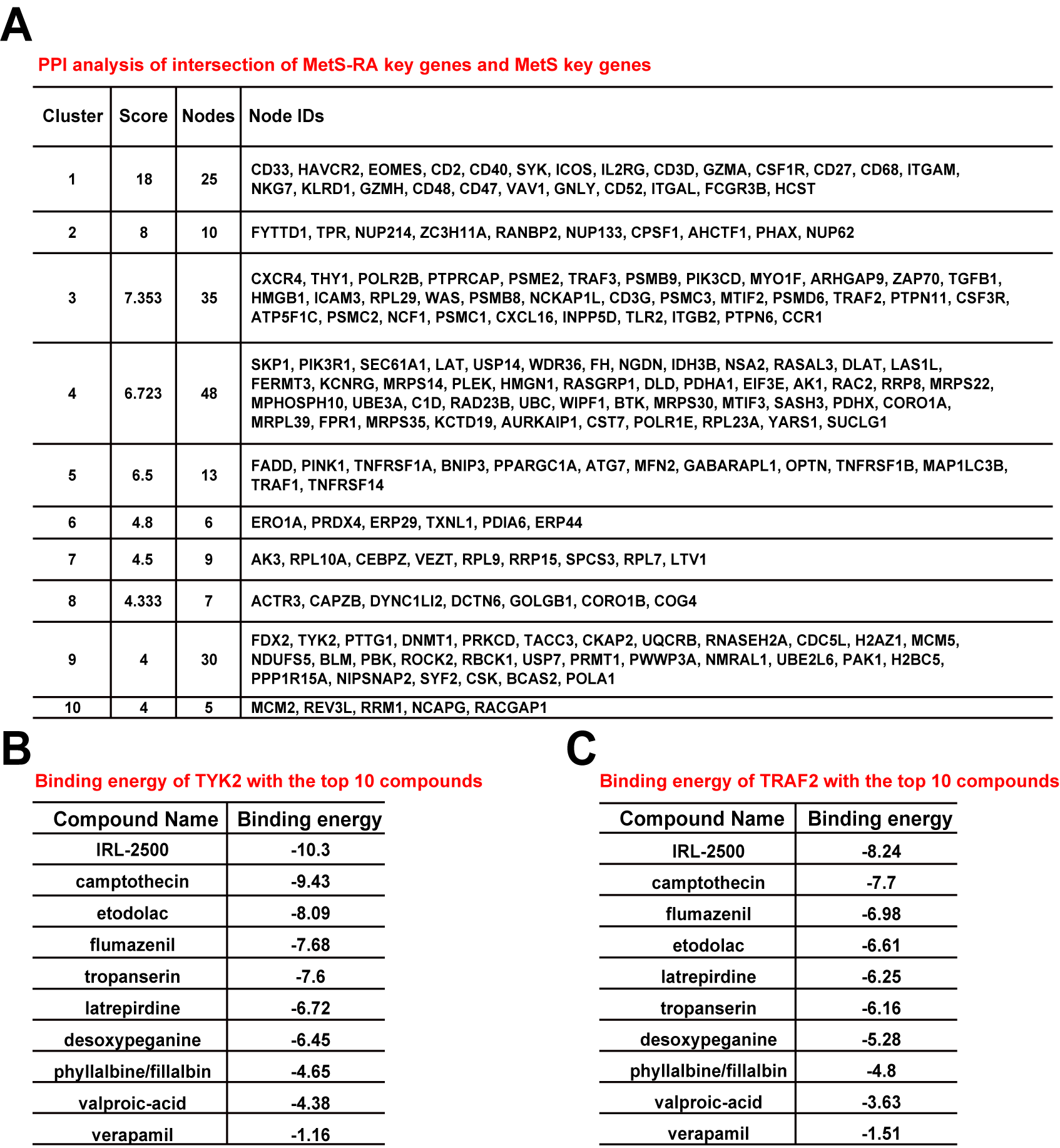


**Supplementary Figure 2**

PPI network cluster analysis and binding energy of molecular docking. (A) Top 10 clusters screened by MCODE function in Cytoscape software. (B) The binding energy of TYK2 with top 10 compounds via molecular docking. (C) The binding energy of TRAF2 with top 10 compounds via molecular docking.

| **Supplementary Table 1** Demographics of RA patients with or without metabolic syndrome | | | |
| --- | --- | --- | --- |
| Group | nMetS | MetS | p value |
| Age (year) | 64.36 ± 6.52 | 62.93 ± 5.87 | 0.7528 |
| Number | 14 | 15 | - |
| Gender (male/female) | 5/17 | 5/35 | 0.7381 |
| BMI (kg/m^2^) | 24.87 ± 2.37 | 29.65 ± 2.27 | **< 0.0001** |
| FBG (mmol/l) | 5.41 ± 0.40 | 6.96 ± 2.22 | 0.0769 |
| Waist circumference (cm) | 91.27 ± 11.52 | 97.93 ± 5.55 | **0.0418** |
| TG (mmol/l) | 1.28 ± 0.34 | 2.49 ± 0.86 | **< 0.0001** |
| HDL (mmol/l) | 1.44 ± 0.23 | 1.13 ± 0.14 | **0.0004** |
| LDL (mmol/l) | 3.30 ± 0.80 | 3.95 ± 0.86 | 0.0551 |
| SBP（mmHg） | 129.10 ± 12.93 | 148.40 ± 18.11 | **0.0027** |
| DBP（mmHg） | 79.57 ± 7.78 | 87.80 ± 9.60 | **0.0100** |
| METS-IR | 35.52. ± 3.00 | 48.20 ± 3.75 | **< 0.0001** |
| Z-score | 0.02 ± 1.20 | 4.84 ± 2.19 | **< 0.0001** |
| Data are number or mean ± SD.  BMI: Body-mass index; FBG: fasting blood glucose; TG: triglyceride; HDL: high density lipoprotein; LDL: low density lipoprotein; SBP: Systolic blood pressure; DBP: diastolic blood pressure; METS-IR: metabolic score for insulin resistance; SD: standard deviation. | | | |

| **Supplementary Table 2** KEGG enrichment analysis of merged RA dataset involved metabolic pathways and metabolic diseases | | |
| --- | --- | --- |
| Term | NES | p value |
| **Type I Diabetes Mellitus** | 1.8463 | **0.0041** |
| **Sulfur Metabolism** | 1.6931 | **0.0093** |
| **Tyrosine Metabolism** | -1.6564 | **0.0125** |
| **Sphingolipid Metabolism** | 1.6991 | **0.025** |
| Phenylalanine Metabolism | -1.4506 | 0.0587 |
| Propanoate Metabolism | -1.5686 | 0.0665 |
| Histidine Metabolism | -1.443 | 0.0722 |
| Linoleic Acid Metabolism | -1.4463 | 0.082 |
| O Glycan Biosynthesis | 1.3781 | 0.092 |
| Retinol Metabolism | -1.3937 | 0.0943 |
| Amino Sugar And Nucleotide Sugar Metabolism | 1.4607 | 0.1035 |
| Glycosphingolipid Biosynthesis Globo Series | 1.4202 | 0.1038 |
| Butanoate Metabolism | -1.3951 | 0.1245 |
| Type II Diabetes Mellitus | 1.2265 | 0.128 |
| Alanine Aspartate And Glutamate Metabolism | -1.3057 | 0.1435 |
| Fatty Acid Metabolism | -1.3613 | 0.1781 |
| Folate Biosynthesis | 1.2741 | 0.1822 |
| Glycosphingolipid Biosynthesis Ganglio Series | 1.3049 | 0.1868 |
| Steroid Hormone Biosynthesis | -1.213 | 0.2082 |
| Taurine And Hypotaurine Metabolism | -1.1832 | 0.247 |
| Galactose Metabolism | 1.1591 | 0.2865 |
| Pyruvate Metabolism | -1.1793 | 0.3097 |
| Ascorbate And Aldarate Metabolism | -1.0919 | 0.3386 |

| **Supplementary Table 3** KEGG enrichment analysis of GSE7307 involved metabolic pathways and metabolic diseases | | |
| --- | --- | --- |
| Term | NES | p value |
| **Butanoate Metabolism** | -1.7385 | **< 0.0001** |
| **Glutathione Metabolism** | -1.5917 | **< 0.0001** |
| **Type I Diabetes Mellitus** | 1.7472 | **0.004** |
| **Histidine Metabolism** | -1.6198 | **0.0179** |
| **Fatty Acid Metabolism** | -1.8035 | **0.0221** |
| **Tyrosine Metabolism** | -1.7243 | **0.0335** |
| **Pyruvate Metabolism** | -1.6485 | **0.044** |
| Linoleic Acid Metabolism | -1.6493 | 0.0549 |
| Propanoate Metabolism | -1.609 | 0.0682 |
| Oxidative Phosphorylation | -1.6071 | 0.0791 |
| Phenylalanine Metabolism | -1.4685 | 0.0958 |
| Tryptophan Metabolism | -1.3122 | 0.1044 |
| Glyoxylate And Dicarboxylate Metabolism | -1.3852 | 0.1111 |
| Ascorbate And Aldarate Metabolism | -1.299 | 0.1362 |
| Glycine Serine And Threonine Metabolism | -1.408 | 0.1443 |
| Glycolysis Gluconeogenesis | -1.3078 | 0.1548 |
| Beta Alanine Metabolism | -1.3134 | 0.1617 |
| Ether Lipid Metabolism | -1.2497 | 0.174 |
| Arginine And Proline Metabolism | -1.1112 | 0.255 |
| Alanine Aspartate And Glutamate Metabolism | -1.1506 | 0.289 |
| Selenoamino Acid Metabolism | -1.0705 | 0.4148 |
| Glycerophospholipid Metabolism | -1.053 | 0.4158 |
| Starch And Sucrose Metabolism | -1.0546 | 0.4308 |
| Biosynthesis Of Unsaturated Fatty Acids | -1.0493 | 0.4334 |
| Arachidonic Acid Metabolism | -1.0263 | 0.437 |
| Purine Metabolism | -1.0617 | 0.4637 |

| **Supplementary Table 4** KEGG enrichment analysis of GSE77298 involved metabolic pathways and metabolic diseases | | |
| --- | --- | --- |
| Term | NES | p value |
| **N Glycan Biosynthesis** | 1.907 | **< 0.0001** |
| **Amino Sugar And Nucleotide Sugar Metabolism** | 1.7095 | **0.0087** |
| **Sulfur Metabolism** | 1.7188 | **0.0117** |
| **Type I Diabetes Mellitus** | 1.7371 | **0.0282** |
| **Galactose Metabolism** | 1.4967 | **0.0428** |
| Sphingolipid Metabolism | 1.521 | 0.0593 |
| Butanoate Metabolism | -1.5185 | 0.0721 |
| Fatty Acid Metabolism | -1.5352 | 0.085 |
| Beta Alanine Metabolism | -1.4845 | 0.0948 |
| Propanoate Metabolism | -1.4935 | 0.101 |
| Glycine Serine And Threonine Metabolism | -1.3623 | 0.105 |
| Ascorbate And Aldarate Metabolism | -1.3456 | 0.116 |
| Steroid Biosynthesis | 1.3575 | 0.122 |
| Folate Biosynthesis | 1.3547 | 0.1223 |
| Histidine Metabolism | -1.3134 | 0.1378 |
| Phenylalanine Metabolism | -1.2939 | 0.1475 |
| Tyrosine Metabolism | -1.2333 | 0.1595 |
| Glycerolipid Metabolism | -1.2501 | 0.1608 |
| Glyoxylate And Dicarboxylate Metabolism | -1.3351 | 0.1718 |
| Fructose And Mannose Metabolism | 1.3274 | 0.1787 |
| Glutathione Metabolism | 1.2214 | 0.2077 |
| Glycosaminoglycan Biosynthesis Keratan Sulfate | 1.2615 | 0.2093 |
| Pyruvate Metabolism | -1.3235 | 0.2172 |
| Biosynthesis Of Unsaturated Fatty Acids | -1.1578 | 0.2635 |
| Pyrimidine Metabolism | 1.1621 | 0.286 |
| Alanine Aspartate And Glutamate Metabolism | -1.1404 | 0.2969 |
| Tryptophan Metabolism | -1.0691 | 0.3824 |
| Alpha Linolenic Acid Metabolism | -1.03 | 0.4313 |

| **Supplementary Table 5** KEGG enrichment analysis of MetS dataset involved metabolic pathways | | | |
| --- | --- | --- | --- |
| Term | NES | p value | Involved Genes |
| **O** **Glycan Biosynthesis** | -1.8745 | **< 0.0001** | *GALNT11/GALNT1/ST3GAL1/GALNT6/GALNT3/GCNT1/GALNT4* |
| **Inositol Phosphate Metabolism** | 1.5626 | **0.0138** | *FIG4/INPP5E/PLCG1/INPP4B/PIK3C2B/ISYNA1/PLCB2/PI4KB/ITPKB/PLCG2/TPI1/PIP4K2C/PIK3CB/PI4K2A/PIK3CG/ALDH6A1/POMGNT1/POMT1/ST3GAL3/B4GALT3/B4GALT1* |
| **N Glycan Biosynthesis** | 1.5118 | **0.0374** | *RPN2/DOLPP1/STT3A/ALG3/DPM3/ALG8/MAN1B1/ST6GAL1/DPAGT1/B4GALT3* |
| **Tryptophan Metabolism** | -1.4986 | **0.0444** | *KYNU/GCDH/DLD/ACAT2/ACMSD/DLST/CYP1B1* |
| Biosynthesis Of Unsaturated Fatty Acids | -1.3586 | 0.1098 | *HACD4/HSD17B12/SCP2/SCD* |
| Pentose Phosphate Pathway | 1.3738 | 0.1365 | *RPE/PGM2/ALDOA/TALDO1/PGLS/PGM1/PFKP/PRPS1/PRPS2/PFKL/G6PD/TKT/ALDOB* |
| Glycolysis Gluconeogenesis | 1.2842 | 0.1393 | *PGAM1/PCK2/PDHA1/ACSS2/ENO3/AKR1A1/LDHB/TPI1/PGAM4/HK1/PGM2/ENO2/ALDOA/PGM1/PFKP* |
| Glycosaminoglycan Biosynthesis Heparan Sulfate | 1.2839 | 0.1434 | *NDST2/EXT1/NDST1* |
| Galactose Metabolism | 1.3626 | 0.1574 | *GALT/GALK1/GLB1/HK1/PGM2/PGM1/B4GALT1/PFKP/PFKL/HK3* |
| Ether Lipid Metabolism | -1.2112 | 0.2115 | *PLA2G2D/PLA2G7/PLB1/CHPT1/GDPD1/PAFAH2* |
| Glycerolipid Metabolism | 1.2084 | 0.2122 | *GPAM/DGKD/GK/MBOAT2/AGPAT5/GPAT3* |
| Glycine Serine And Threonine Metabolism | -1.1942 | 0.2173 | *PGAM1/GRHPR/PGAM4* |
| Purine Metabolism | 1.1177 | 0.2419 | *AK3/NT5C2/ENPP4/DCK/ADK/PDE4B/PDE9A/PAPSS2/ADCY3/NME7/PNP/HPRT1/PDE1B/RRM1/RRM2B/ENTPD4/NME2/ADCY2/PDE3B/PDE4D* |
| Glycerophospholipid Metabolism | -1.1056 | 0.2895 | *GPAM/DGKD/CHPT1/CHKA/LYPLA1/MBOAT2/AGPAT5/GPAT3* |
| Amino Sugar And Nucleotide Sugar Metabolism | 1.0595 | 0.3446 | *GALT/UGDH/NANS/GALK1/GMPPA/NAGK/UAP1/HK1/PGM2* |
| Starch And Sucrose Metabolism | -1.0589 | 0.3708 | *PGM2L1/GYS1/GAA/UGP2/HK2/AMY1A/AMY1B* |
